# ASO Author Reflections: When a New Standard Becomes the Standard: Translating Neoadjuvant Chemo-Immunotherapy for Resectable NSCLC Into Real-World Practice

**DOI:** 10.1245/s10434-026-20473-y

**Published:** 2026-08-25

**Authors:** Jiri Vachtenheim Jr, Fernando Casas-Mendez, Rene Novysedlak, Janis Tavandzis, Boris Sepesi, Robert Lischke

**Affiliations:** 1 3rd Department of Surgery, First Faculty of Medicine, Charles University and University Hospital Motol and Homolka, Prague, Czech Republic; 2 Pneumology and Oncology Departments, Second Faculty of Medicine, Charles University and Center for Thoracic Oncology and Surgery, University Hospital Motol and Homolka, Prague, Czech Republic; 3 Sarah Cannon Cancer Network, Swedish Medical Center, Englewood, CO USA

## Past

For decades, up-front surgery has represented an important cornerstone of curative-intent treatment for medically fit patients with resectable non-small cell lung cancer (NSCLC). The advent of modern systemic therapy (targeted therapy and immunotherapy) has transformed the treatment landscape of various solid tumors, and oncology has entered an era in which practice-changing therapies emerge at an unprecedented pace. Immune check-point inhibitors (ICI) have revolutionized the treatment of unresectable NSCLC. After proving efficacy and safety in the metastatic setting, ICI have been investigated by several phase II, followed by seminal phase III clinical trials in neoadjuvant and perioperative setting for resectable NSCLC treatment.

Following the pivotal CheckMate816 trial published in 2023, neoadjuvant nivolumab-based chemo-immunotherapy demonstrated significantly improved event-free survival and pathological complete response (pCR) compared with neoadjuvant chemotherapy alone and was rapidly accepted as a new standard of care for selected patients with resectable NSCLC.^1,2^ However, the implementation of this new treatment paradigm into various healthcare systems around the world brings its own unique challenges. It requires experienced multidisciplinary teams, institutional coordination, surgical expertise, and confidence that comparable outcomes can be reproduced outside the highly controlled environment of randomized clinical trials. The evidence from various national healthcare systems describing how this therapeutic strategy performs has been scarce.

## Present

Our nationwide prospective multicenter study examines and captures this critical phase of clinical transformation. By prospectively including all accredited thoracic surgical oncology centers in the Czech Republic, we evaluated the adoption of neoadjuvant chemo-immunotherapy during its early implementation from a surgical perspective.^3^

The results are encouraging. Although technical operative difficulties (mainly significant hilar fibrosis and lymphadenopathy) were frequently encountered during pulmonary resection, R0 resection was achieved in nearly all operated patients, while perioperative morbidity and mortality remained comparable to historical trends and pCR was 40.6% in the overall (intention-to-treat) population.^3^

Our study represents more than a report of surgical or pathological outcomes. It provides evidence that nationwide implementation of a complex multidisciplinary treatment strategy is feasible, safe, and successfully integrated into routine practice. The real-world setting in our study also reveals potential practical challenges that must be addressed to ensure not only comparability across future studies, but also consistent implementation of this treatment strategy across different healthcare systems.

## Future

Clinical trials establish new standards of care. Prospective nationwide implementation studies further demonstrate whether entire clinical communities are ready and able to deliver them effectively to their patients. From a surgical perspective, future efforts should focus on patient selection, standardizing pathological assessment, refining perioperative management, and identifying predictors of surgical complexity, long-term benefits, and patient-reported outcomes.

As perioperative treatment of resectable NSCLC is rapidly entering an era of increasing complexity (disease biology and personalized medicine), future advances and outcomes will depend not solely on the novel therapeutic agents, but also on ensuring that these increasingly complex treatment pathways are delivered within experienced multidisciplinary centers for thoracic oncology with high-quality and high-volume surgical care.^4^
